# A Rare Manifestation of a Presumed Non-Osteophilic Brain Neoplasm: Extensive Axial Skeletal Metastases From Glioblastoma With Primitive Neuronal Components

**DOI:** 10.3389/fonc.2021.760697

**Published:** 2021-11-02

**Authors:** Tianhua Rong, Wanjing Zou, Xiaoguang Qiu, Wei Cui, Duo Zhang, Bingxuan Wu, Zhuang Kang, Wenbin Li, Baoge Liu

**Affiliations:** ^1^ Department of Orthopaedic Surgery, Spine Center, Beijing Tiantan Hospital, Capital Medical University, Beijing, China; ^2^ National Clinical Research Center for Orthopedics, Sports Medicine & Rehabilitation, Beijing, China; ^3^ Basic and Translational Medicine Center, China National Clinical Research Center for Neurological Diseases, Beijing, China; ^4^ Department of Neuropathology, Beijing Neurosurgical Institute, Capital Medical University, Beijing, China; ^5^ Department of Radiation Oncology, Beijing Tiantan Hospital, Capital Medical University, Beijing, China; ^6^ Department of Neuro-Oncology, Cancer Center, Beijing Tiantan Hospital, Capital Medical University, Beijing, China

**Keywords:** glioblastoma multiforme, primitive neuronal component, extracranial metastasis, treatment, prognosis, pathology

## Abstract

**Background:**

Glioblastoma multiforme (GBM) is the most common malignant tumor of the central nervous system. GBM with primitive neuronal component (GBM-PNC) is an aggressive variant identified in 0.5% of GBMs. Extracranial metastasis from GBM-PNC is a rare and challenging situation.

**Methods:**

A special case of early-onset GBM with systemic bone metastasis was enrolled. Clinical data, including patient characteristics, disease course, and serial radiological images were retrieved and analyzed. Tumor tissues were obtained by surgical resections and were made into formalin-fixed paraffin-embedded sections. Histopathological examinations and genetic testing were performed for both the primary and metastatic tumor specimens.

**Results:**

A 20-year-old man suffered from GBM with acute intratumoral hemorrhage of the left temporal lobe. He was treated by gross total resection and chemoradiotherapy following the Stupp protocol. Seven months later, he returned with a five-week history of progressive neck pain and unsteady gait. The radiographic examinations identified vertebral collapse at C4 and C6. Similar osteolytic lesions were also observed at the thoracolumbar spine, pelvic, and left femur. Anterior spondylectomy of C4 and C6 was performed. The resected vertebral bodies were infiltrated with greyish, soft, and ill-defined tumor tissue. One month later, he developed mechanical low-back pain and paraplegia caused by thoracolumbar metastases. Another spine surgery was performed, including T10 total en-bloc spondylectomy, T7-9, L2-3, and L5-S1 laminectomy. After the operation, the patient’s neurological function and spinal stability remained stable. However, he finally succumbed to the rapidly increased tumor burden and died 15 months from onset because of cachexia and multiple organ failure. In addition to typical GBM morphology, the histological examinations identified monomorphic small-round cells with positive immunohistochemical staining of synaptophysin and CD99, indicating the coexistence of PNC. The next-generation sequencing detected pathogenic mutations in TP53 and DNMT3A. Based on above findings, a confirmed diagnosis of systemic metastases from GBM-PNC (IDH-wild type, WHO grade IV) was made.

**Conclusions:**

The present case highlights the occurrence and severity of extensive axial skeletal metastases from GBM-PNC. This rare variant of GBM requires aggressive multimodal treatment including surgery and chemoradiotherapy targeting PNC. The pathological screening of PNC is recommended in patients with early-onset GBM and intratumoral hemorrhage. Surgery for spinal metastasis is appropriate in patients with chemoradioresistance and relatively good general status, with the objectives of restoring spinal stability and relieving spinal cord compression.

## Introduction

Glioblastoma multiforme (GBM) is the most common malignant tumor of the central nervous system (CNS) and comprises approximately 48.6% of primary malignant brain tumors and approximately 57.7% of all gliomas (1). Recently, with a better understanding of the biological behavior of GBM, the long-held dogma that GBM does not metastasize outside the brain has been overturned. A growing number of reports have documented extracranial metastasis in GBM (2). Though highly invasive, the extracranial metastases of GBM are rare, with an incidence estimated at 0.4%-2% (3). The majority of extracranial metastases occur after craniotomy, but spontaneous metastasis has also been documented (4). Usually, extracranial metastases are not discovered until the very advanced stage of the course of GBM, and the median duration from detection of extracranial metastases to death is 1.5 months (range: 0–14 months) (2). The pathogenesis of extracranial metastasis of GBM remains unclear, and effective treatment strategies are lacking. Further case studies are therefore needed to better understand disease processes.

GBM with primitive neuronal component (GBM-PNC) is an emerging variant of GBM introduced in the new WHO classification of tumors of the CNS in 2016 and was renamed from “glioblastoma with primitive neuroectodermal tumor (PNET)-like component” (5). On histology, PNC is detected in about 0.5% of GBM cases (6). GBM-PNC is widely regarded as an aggressive malignant tumor with a high risk of metastasis and short survival (7). Most previous reports on this rare entity are case reports or case series with limited sample size, and the documentation of extracranial metastases from GBM-PNC is scarce. Consequently, the diagnosis and treatment of GBM-PNC are not yet established, and extracranial metastases from this tumor pose a formidable challenge to clinicians.

Here, we describe a case of GBM-PNC with extensive axial skeletal extracranial metastases without local recurrence, treated with three surgeries and chemoradiotherapy. The particularity of clinical manifestations and the results of histological and genetic examinations are briefly discussed.

## Methods

### Patient Selection and Clinical Data

A special case of histopathologically confirmed early-onset supratentorial GBM with systemic bone metastasis was enrolled. Information, including patient characteristics, disease course, and serial radiological images was retrieved from the medical records and picture archiving and communication systems of our hospital. Treatment decisions were made by a multidisciplinary GBM advisory council (organized by the senior authors BL, WL, and XQ). Informed consent was obtained from the patient’s parents for participation in the present study. Ethical approval was obtained from the Institutional Review Board of Beijing Tiantan Hospital, Capital Medical University, Beijing, China (KY2014-025-02).

### Neuropathological Examination and Genetic Testing

Tumor tissues were obtained from the patient by surgical resections, which were made into formalin-fixed paraffin-embedded sections. The morphology of tumor cells was evaluated by hematoxylin-eosin staining. Further immunohistochemical analyses were performed with antibodies against glial fibrillary acidic protein (GFAP, OriGene Technologies, USA, 1:50), oligodendrocyte transcription factor 2 (Olig-2, OriGene, 1:200), Synaptophysin (Syn, OriGene, 1:100), and CD99 (OriGene, 1:150). The proliferation index was measured by Ki-67 labeling (OriGene, 1:50). The DNA was extracted from paraffin sections for molecular diagnosis. Sanger sequencing and next generation sequencing (targeted panel and whole exome) were applied in the primary and metastatic tumor specimens respectively. The response to the immune checkpoint therapy was predicted by both sequencing and immunofluorescence. A protein-protein interaction network was constructed with the pathogenic mutant genes in this patient and previously reported GBM-associated genes.

## Results

### Clinical History

**Figure 1:**
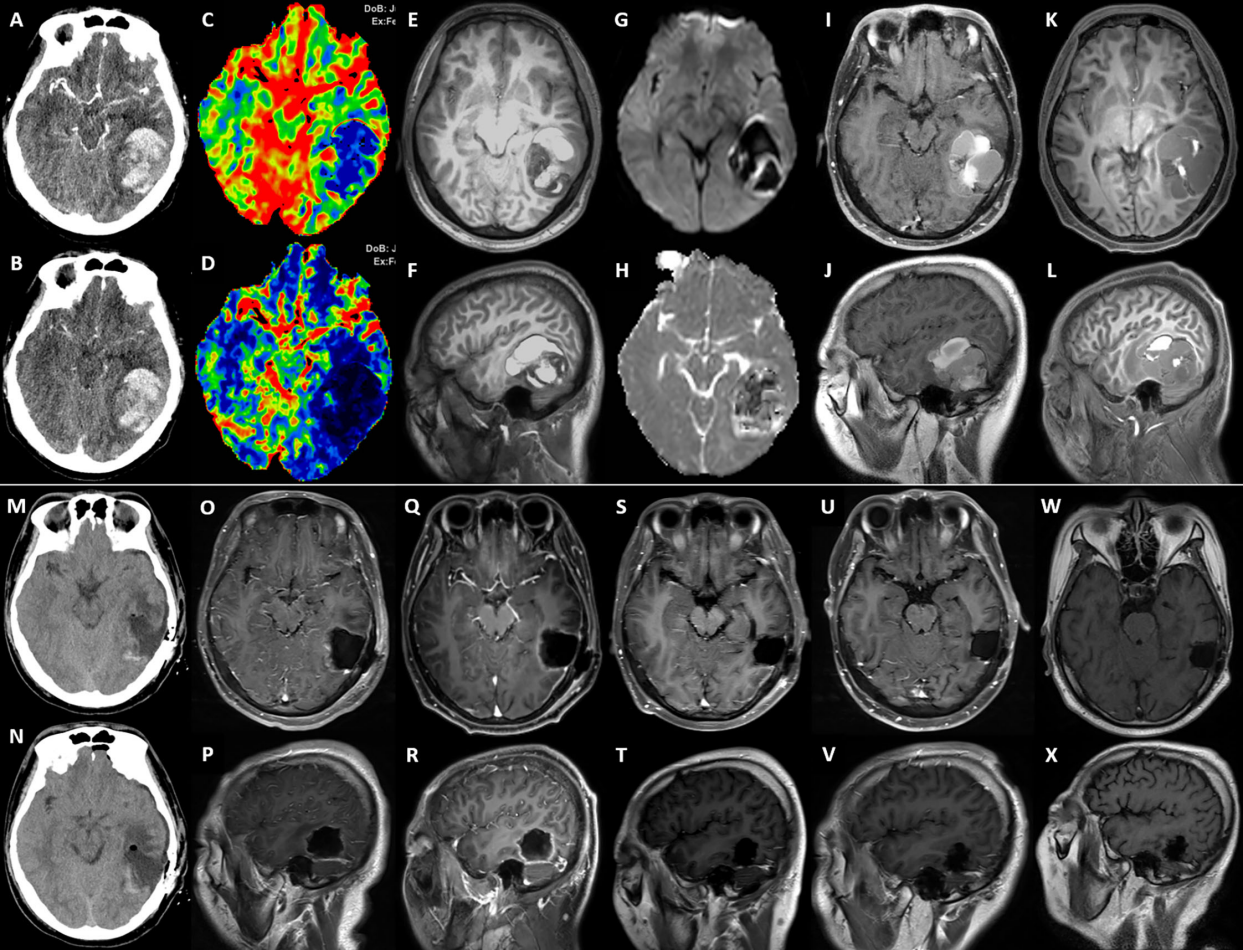
The serial imaging of the intracranial lesion. **(A–L)** are preoperative images, and **(M–X)** are postoperative images. **(A, B)** The arterial phase and venous phase of cerebral CT angiography taken at onset (9^th^ Feb. 2020). **(C, D)** The volume-based and flow-based CT perfusion images showed relatively low perfusion status of the lesion. **(E, F)** The T1 weighted MRI imaging with the three-dimensional magnetization-prepared rapid acquisition with gradient echo (T1WI-3D-MPRAGE) (10^th^ Feb.) showed hemorrhagic cystic supratentorial neoplasm. **(G, H)** The b1000 and apparent diffusion coefficient images from diffusion-weighted imaging showed restricted diffusion within the lesion. **(I, J)** The T1WI MRI with contrast on 30^th^ Mar. identified that the hematoma was partially absorbed. **(K, L)** The preoperative T1WI-3D-MPRAGE MRI on 10^th^ Apr. showed significant absorption of the hemorrhage. **(M, N)** Postoperative CT scans, **(O, P)**, postoperative T1WI MRI with contrast, both demonstrated a gross total resection of the left temporal lesion. The periodical follow-up T1WI MRIs with contrast, including **(Q, R)** (29^th^ Apr.), **(S, T)** (17^th^ Jul.), **(U, V)** (28^th^ Oct., before cervical spine surgery), and **(W, X)** (25^th^ Jan. 2021, right after thoracolumbar spine surgery, and 9 months after the neurosurgery), observed no sign of local recurrence.

On February 8, 2020, a 20-year-old man came to our hospital with chief complaints of headache, vomiting, diplopia, alexia, and transient amnesia for 2 hours. Brain magnetic resonance imaging (MRI) showed a 6×5-cm neoplasm with intratumoral hemorrhage of the left temporal lobe (**Figures 1A–L**). The lesion had a well-defined boundary without surrounding vasogenic edema, and reduced diffusion was observed (**Figures 1G, H**). Computed tomography (CT) with contrast identified a hemorrhagic lesion consistent with MRI, which showed hypo-perfusion change on CT perfusion imaging (**Figures 1C, D**). After 3 weeks of close observation and supportive treatment, the patient’s general status became stable, and he was discharged on February 28. Re-examination of brain MRI on March 30 showed significant absorption of intralesional hematoma (**Figures 1K, L**). He was then re-admitted to the Department of Neurosurgery, and a craniotomy was scheduled on April 13, during which gross total resection of a 3×3×4-cm tumor was performed (**Figures 1M–P**). The tumor tissue was grayish-yellow and very soft, within which a 0.5-cm-cystic structure with yellow fluid was observed. The patient recovered well and was discharged on April 26. At the Department of Radiation Oncology, he received focal radiotherapy of 60 Gy in 30 fractions over 30 days with concurrent temozolomide (TMZ) chemotherapy (75 mg/m^2^), followed by maintenance TMZ for 6 cycles (150 mg/m^2^ for the first cycle and 200mg/m2 for the next 5 cycles). This standard-of-care chemoradiotherapy was well tolerated. The patient experienced a 6-month adverse event-free survival after neurosurgery. His follow-up MRIs from immediately to 9 months after operation identified no sign of local recurrence (**Figures 1Q–X**).

**Figure 2:**
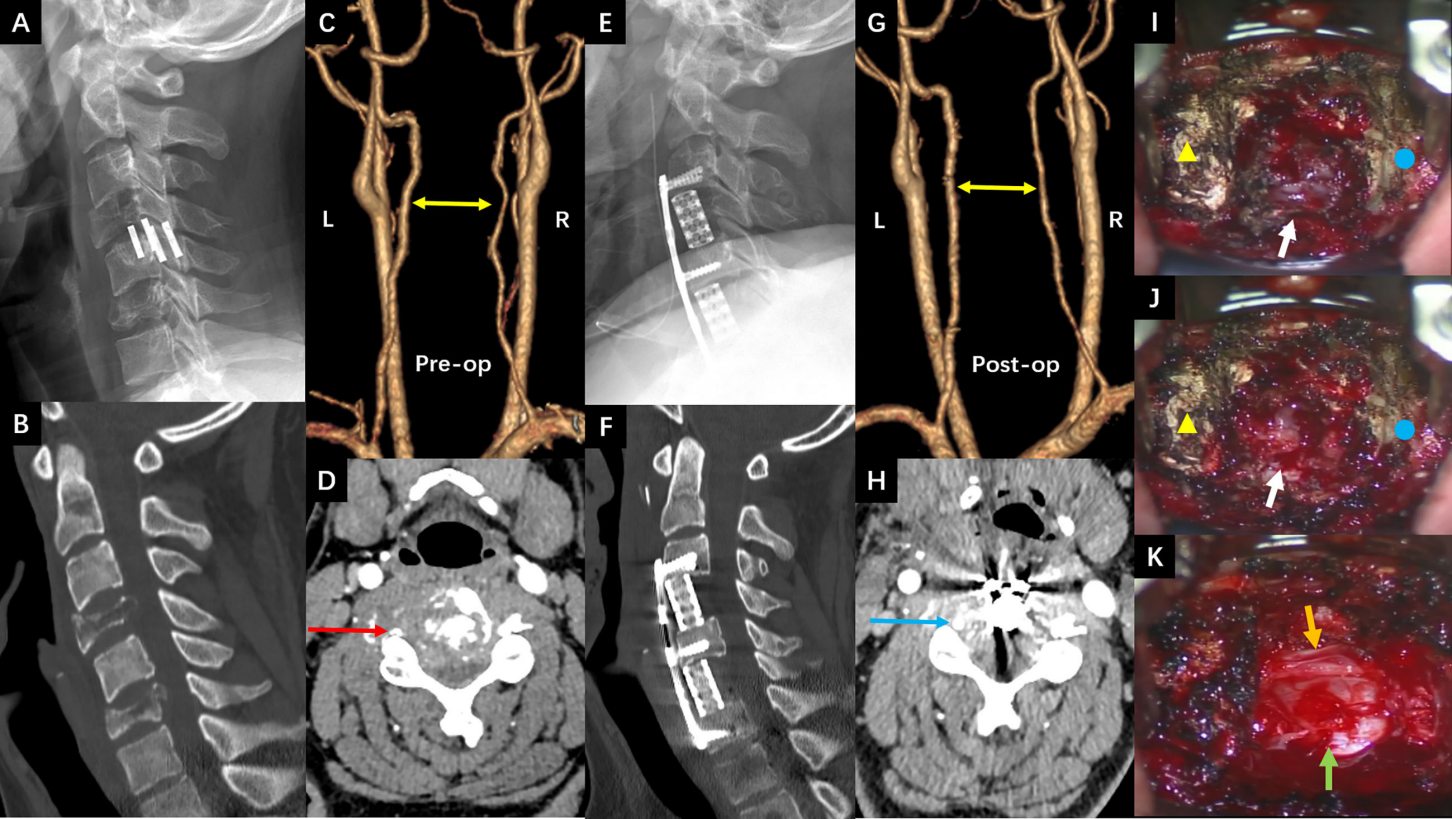
**(A, B)** The sagittal view of preoperative cervical spine radiograph and CT scan showed osteolytic lesions and vertebral collapse at C4 and C6 (2^nd^ Dec. 2020). **(C, D)** The preoperative CT angiography showed the tortuous vertebral arteries of both sides (yellow double arrow) and tumor compression on the right vertebral artery (red arrow) (16^th^ Dec.). **(E, F)** The sagittal view of postoperative cervical spine radiograph and CT scan showed good implant position and complete spondylectomy of C4 and C6 (18^th^ Dec.). **(G, H)** The postoperative CT angiography showed that the vertebral arteries became straight and well filled after removing the compression from metastatic tumors (yellow double arrow and blue arrow) (23^rd^ Dec.). **(I–K)** Intraoperative pictures under surgical microscope before, during and after the C4 spondylectomy (yellow triangle: C3 vertebral body; blue circle: C5 vertebral body; white arrow: C4 vertebral body infiltrated with tumor tissue; green arrow: dural sac; Orange arrow: left vertebral artery).

**Figure 3:**
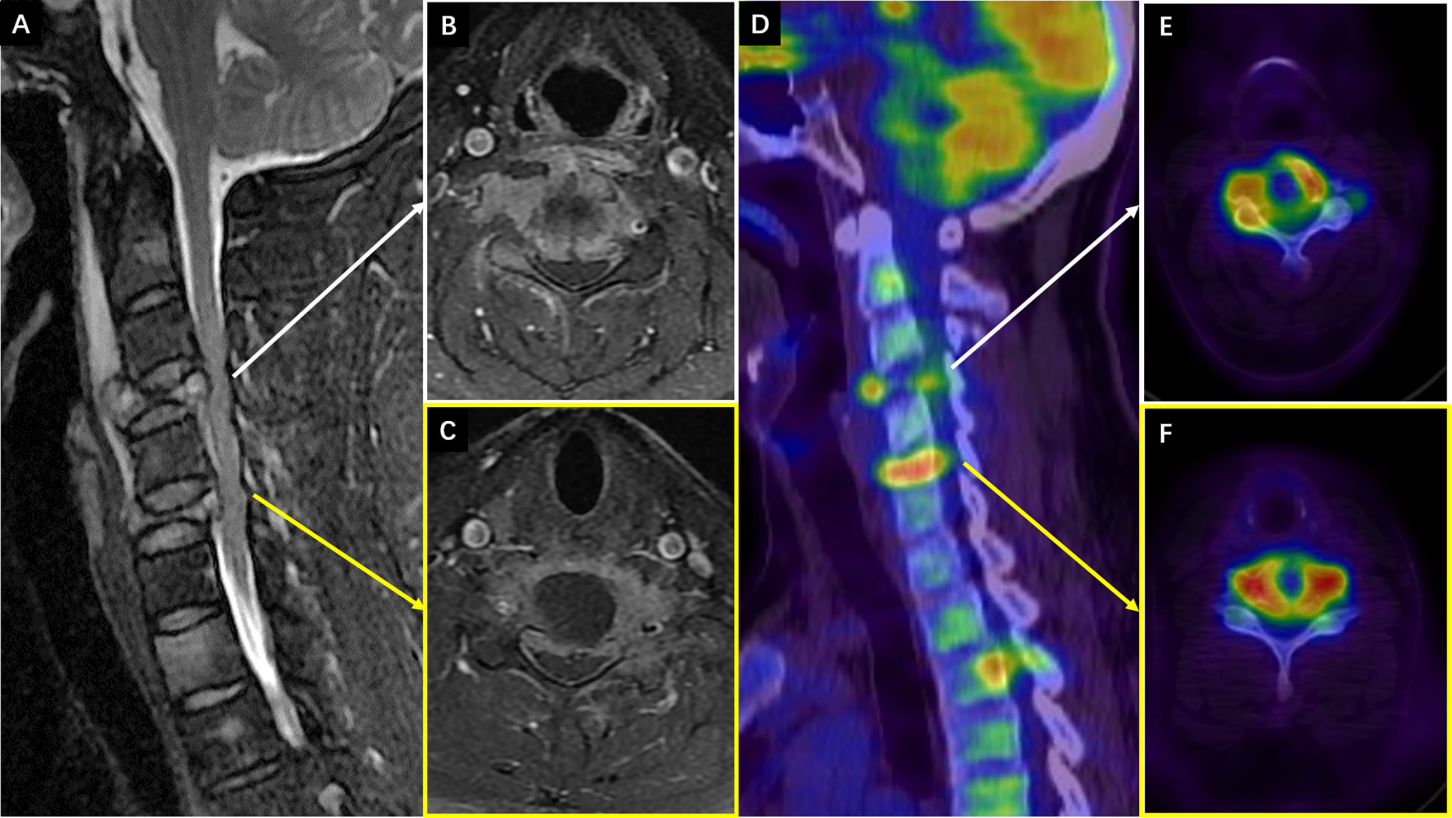
**(A–C)** The preoperative cervical spine MRI (8^th^ Dec. 2020). **(A)** Sagittal T2 weighted imaging showed vertebral collapse at C4 and C6 that compressed the spinal cord. **(B, C)** The axial T1 weighted imaging with contrast showed irregular enhancement of the metastatic lesions at C4 and C6 respectively, and compression of the spinal cord and vertebral arteries (right side at C4 and left side at C6). **(D–F)** The preoperative PET/CT images with views and levels corresponding to the MRI showed increased radioactive uptake at C4 and C6 (22^nd^ Dec.).

The third hospitalization was on December 1. The patient was referred to the Department of Orthopaedic Surgery with chief complaints of severe mechanical neck pain for 5 weeks [visual analog scale (VAS): 80/100 mm] and unsteady gait for 1 week. Physical examination identified extensive tenderness of the neck and hypermyotonia in both lower limbs. Vertebral collapse and compression of the vertebral artery due to the osteolytic lesions at C4 and C6 was identified on plain radiographs and CT scans (**Figures 2A–D**). On cervical spine MRI with contrast, the metastatic lesions were enhanced irregularly, and the transverse foramens and the dural sac were compressed severely (**Figures 3A–C**). Positron emission tomography/CT (PET/CT) identified extensive skeletal metastases with destruction of cancellous bone at multiple vertebrae, right clavicle, left 4^th^ rib, pelvis, and greater trochanter of the left femur (**Figures 3D–F** and **5A–C**). The revised Tokuhashi score (8) and Tomita score (9) were 9 and 8 respectively, and the Spine Instability Neoplastic Score (SINS) (10) of C4 and C6 were both 14. Cervical spine surgery was performed to restore cervical stability and preserve spinal cord function. The patient received C4 and C6 spondylectomy, anterior cervical reconstruction with titanium mesh and autologous iliac bone graft, and plate-screw fixation on December 17. The resected vertebral body was infiltrated with grayish, fragile and ill-defined tumor tissues (**Figures 2I, J**). Post-operative images demonstrated a good implant position and adequate tumor resection without damaging the vertebral artery (**Figures 2E–H**). The patient recovered uneventfully and regained daily-living ability 1 week after operation. The VAS of neck pain decreased from 80 mm to 20 mm.

**Figure 4:**
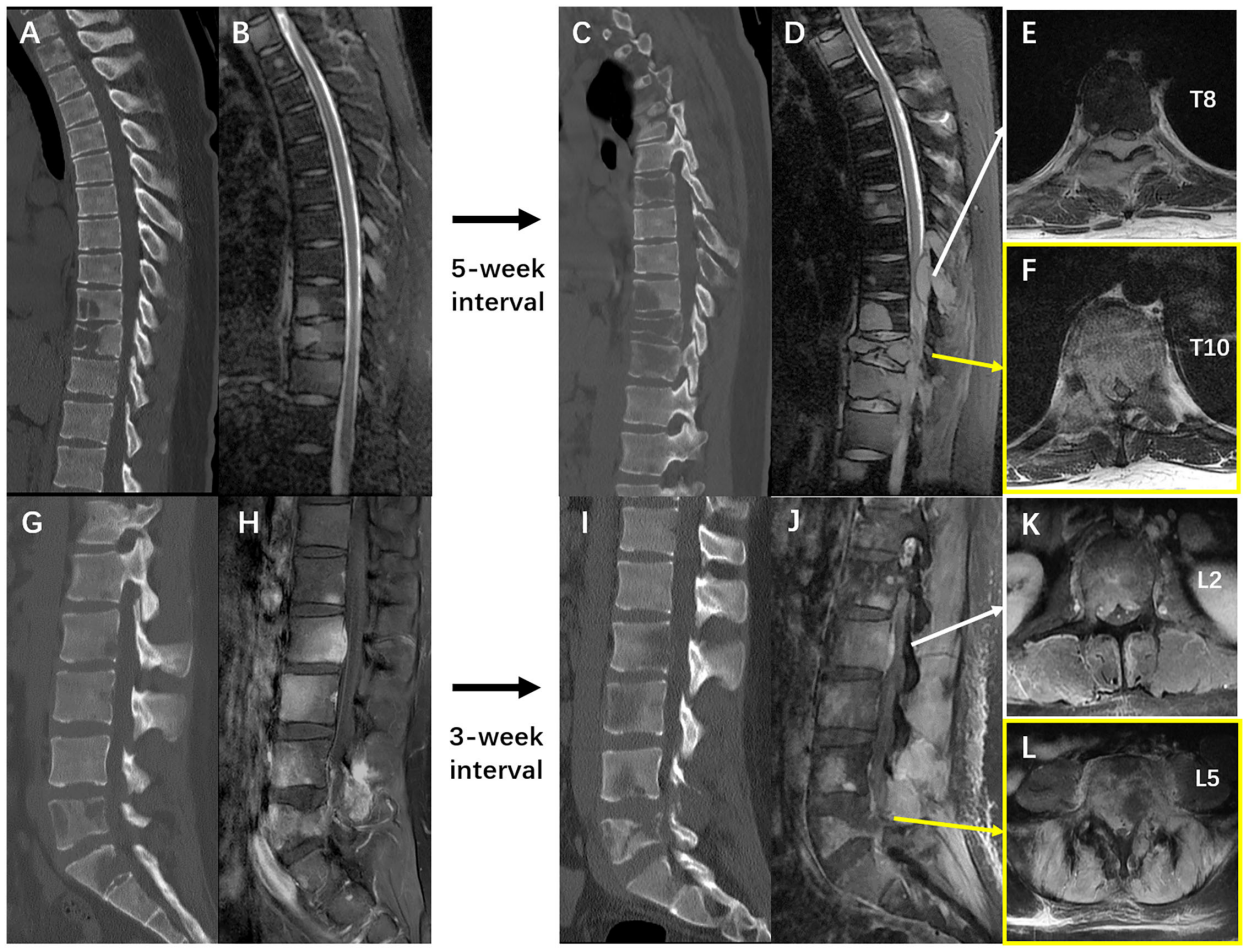
**(A, B)** The sagittal films of thoracic spine CT scan and T2 weighted MRI imaging on 2^nd^ Dec. 2020 and 4^th^ Dec. 2020 respectively. **(C, D)** The sagittal films of thoracic spine CT scan and T2 weighted MRI imaging on 11^th^ Jan. 2021 and 12^th^ Jan. 2021 respectively. **(E, F)** The T1 weighted enhanced imaging of the thoracic spine MRI performed on 12^th^ Jan. 2021 showed severe metastatic epidural spinal cord compression at T8 and T10. **(G, H)**, the sagittal films of lumbar spine CT scan and T1 weighted MRI imaging with contrast on 23^rd^ Dec. 2020 and 22^nd^ Dec. 2020 respectively. **(I, J)** The sagittal films of thoracic spine CT scan and T1 weighted MRI imaging with contrast on 8^th^ Jan. 2021 and 12^th^ Jan. 2021 respectively. **(K, L)** The T1 weighted enhanced imaging of the thoracic spine MRI performed on 12^th^ Jan. 2021 showed severe metastatic epidural spinal cord compression at L2 and L5, and soft tissue invasion in lumbar paraspinal muscles. These images collectively illustrated an extremely rapid progression of the extensive spinal metastases from GBM-PNC.

**Figure 5:**
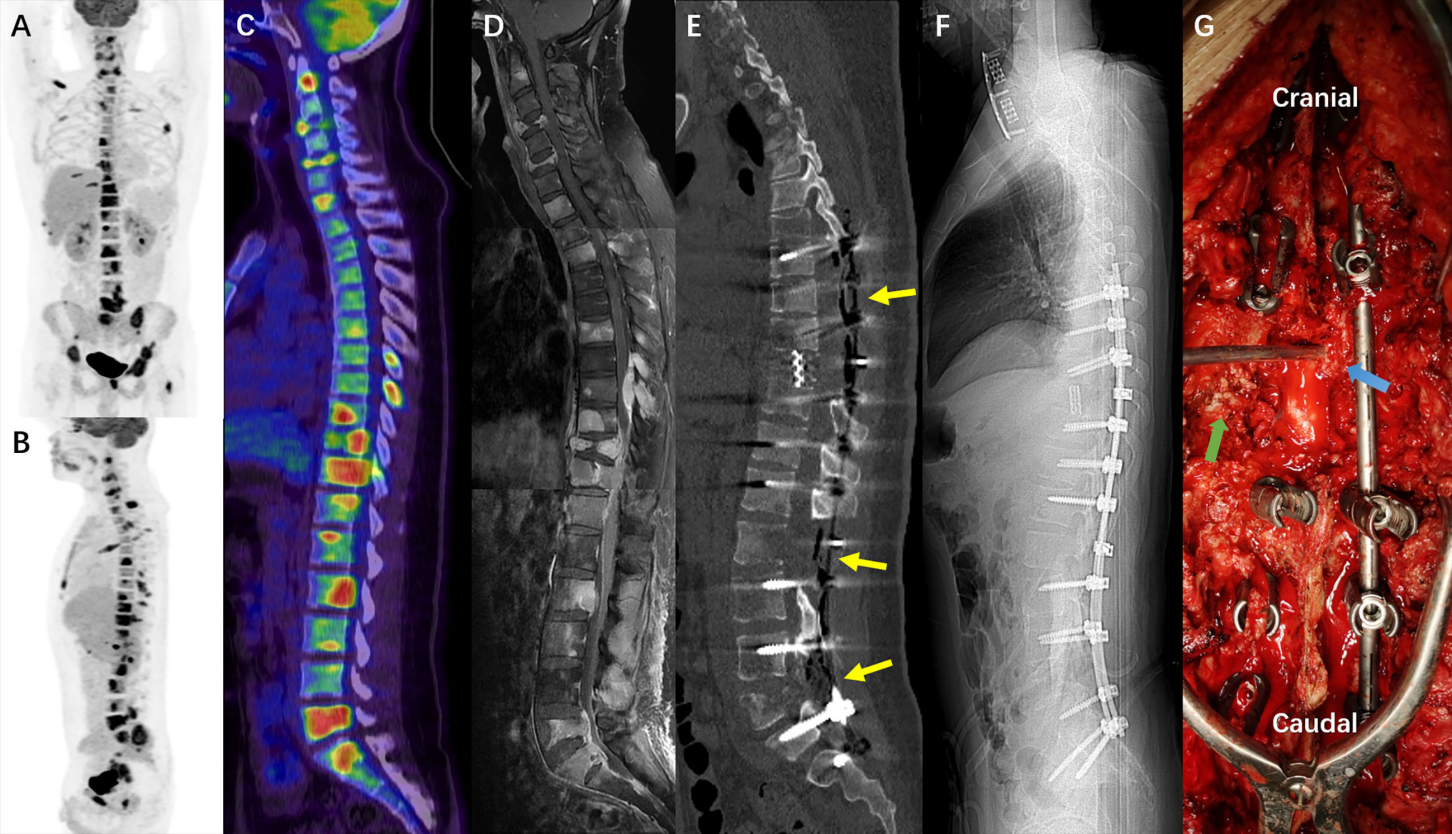
**(A–C)** The PET/CT images (22^nd^ Dec. 2020) showed extensive osseous metastases including multilevel vertebrae, right clavicle, left 4^th^ rib, pelvis, and the great trochanter of left femur. **(D)** The mosaic image of cervical, thoracic and lumbar MRI with contrast, showing extensive axial skeletal metastases corresponding to the PET/CT, and intraspinal drop metastases compressing the spinal cord. **(E, F)**, the sagittal reconstruction and scout scan of postoperative all spine CT demonstrated the multilevel laminectomy (T7-9, L2-3, and L5-S1) and T10 total spondylectomy with titanium mesh reconstruction (18^th^ Jan. 2021). **(G)**, the intraoperative picture during T10 spondylectomy showed metastatic tumor tissues in the paraspinal muscles (green arrow) and the epidural space of the thoracic canal (blue arrow).

Unfortunately, after a moderate sudden external force, the patient developed mechanical low back pain (VAS: 80 mm) from January 5, 2021, with gradual onset of motor and sensory deficits in both lower limbs. On January 8, his neurological condition further deteriorated to complete paraplegia with bladder and bowel dysfunction. Comparisons between MRIs and CTs from two time points with a 3–5-week interval indicated rapid progression of thoracolumbar metastases and vertebral collapse of T10 and L5 (**Figures 4A–D, G–J**). Axial views on enhanced MRI showed severe multi-level compression of the dural sac at T8, T10, L2, and L5. The SINS of T10 and L5 were 14 and 13, respectively (**Figures 4E, F, K, L**). He was admitted again, and another surgical intervention for the thoracolumbar spine was performed on January 15, comprising T10 total en-bloc spondylectomy, T7-9, L2-3, and L5-S1 laminectomy, tumor debulking, and spinal-pelvic fixation (**Figure 5**). After the operation, the level of neurological deficit improved from T8 to T10. The patient developed tumor-induced bone pain and remittent fever, which were treated with analgesics, diphosphonate, nutrition support, and anti-infection therapy. His condition was then improved and the last brain MRI was performed on January 25, 9 months after the neurosurgery, which identified no local recurrence.

The patient’s neurological function and spinal stability remained stable. However, because of the rapidly increasing tumor burden, his general status deteriorated progressively, and he was referred to a local hospital for end-stage treatment on February 17. He developed weakness of the upper limbs and refractory dyspnea caused by the recurrence of cervical metastases and multi-level spinal cord compression identified on repeat MRI. He suffered disturbance of consciousness and was intubated for the last month of his life, and finally died on May 6, 15 months from onset, because of cachexia and multiple organ failure.

### Pathological and Genetic Findings

**Figure 6:**
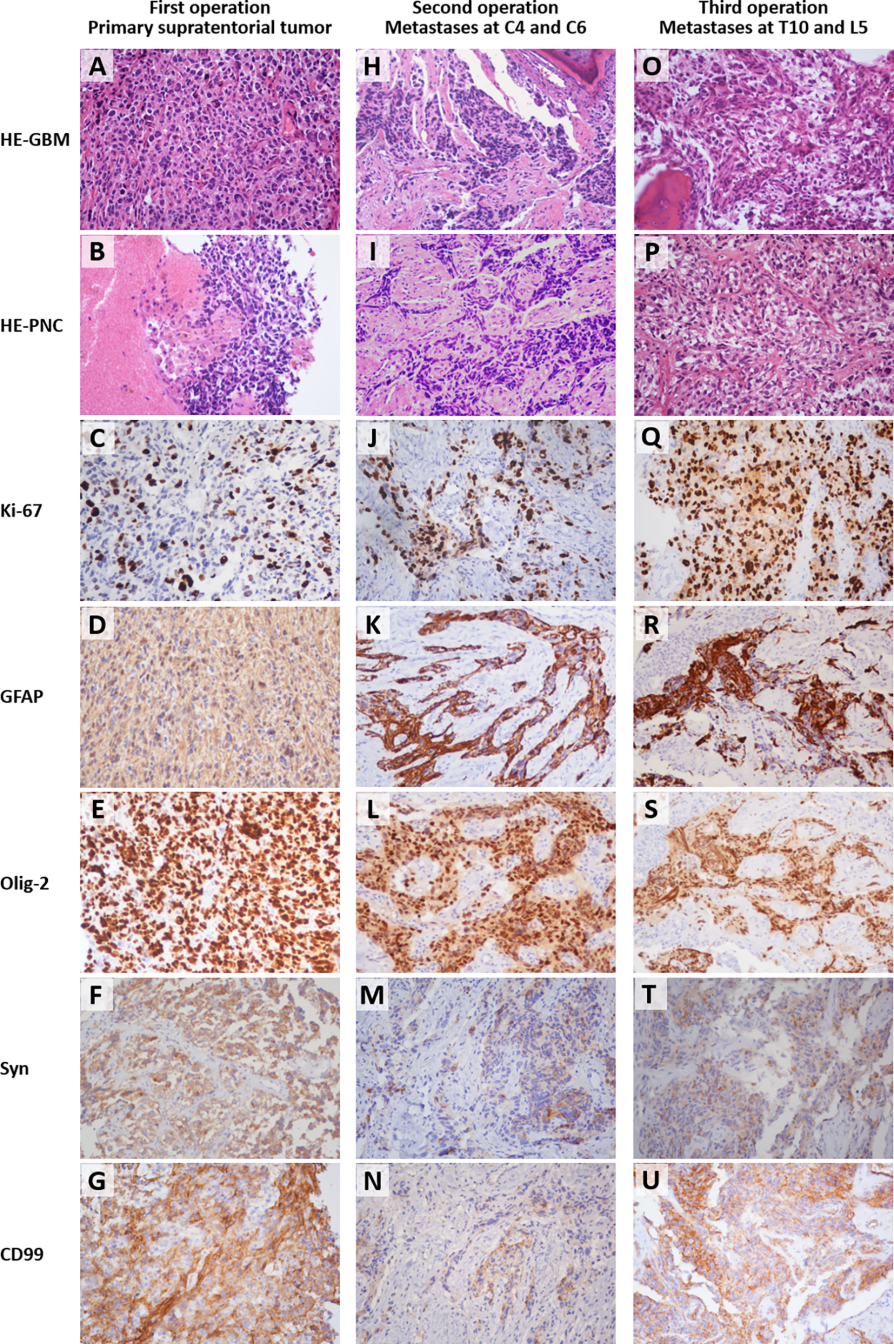
Histological images (200 × magnification) of the formalin-fixed paraffin-embedded sections from the primary supratentorial tumor **(A–G)**, cervical spine metastases **(H–N)**, and thoracolumbar metastases **(O–U)**. The types of staining and sources of the specimens were labeled on the left side and the top, respectively. The osseous tissues were included in the right upper corner of field H and left lower corner of field O. HE, hematoxylin and eosin staining; GBM, glioblastoma multiforme, refers to the field with typical GBM characteristics; PNC, primitive neuronal component, refers to the field with primitive neuroectodermal tumor-like features; GFAP, glial fibrillary acidic protein; Olig-2, oligodendrocyte transcription factor 2; Syn, synaptophysin; CD99, cluster of differentiation 99 (MIC2). The small round blue cells under HE **(B, I, P)** and the positive immunohistochemical staining of Syn **(F, M, T)** indicated the existence of PNC. The Ki-67 labeling index ranged from 40%-80% **(C, J, Q)**.

The initial pathological examination of the intracranial tumor reported anaplastic astrocytoma with partial transformation to GBM (WHO III-IV). However, instead of using specific glioma classification, the second histopathologic analysis of the metastatic tumor tissues from the C4 and C6 vertebral bodies reported malignant tumors of primitive neuroectodermal origin with glial differentiation, given the unusual biphasic histology. The third histopathologic examination of para- and intra-vertebral tissues at T10 and L5 identified multiple features including anaplastic astrocytoma, GBM, and areas of high cellular monomorphism. To resolve the inconsistency and make an accurate differential diagnosis between glial and neuronal origin, a review of all the specimens from the three operations was performed, along with immunohistochemical staining of Syn and CD99 (**Figures 6A–U**).

In histological sections from the primary supratentorial tumor, a malignant glial morphology was observed, with a mixture of both better-differentiated neoplastic astrocytes and poorly-differentiated pleomorphic cells, including small, granular, and giant cells (**Figure 6A**). Monomorphic small, round, blue cells with small hyperchromatic nuclei, namely the PNET-like component or PNC, were only observed in a few areas (**Figure 6B**). As for the vertebral metastases, the PNC accounted for a larger proportion, and even became predominant in some high-magnification (200×) fields (**Figures 6I, P**). The layouts of GBM and PNC elements were in an intermixed pattern with partially merged demarcation, and Homer–Wright rosettes were not observed. The Ki-67 labeling index of the three specimens ranged from 40% to 80%, significantly higher than typical GBM (**Figures 6C, J, Q**) (11). The specimens were consistently positive for Syn and CD99, which suggests the presence of PNC (**Figures 6F, G, M, N, T, U**) (7, 12).

**Figure 7:**
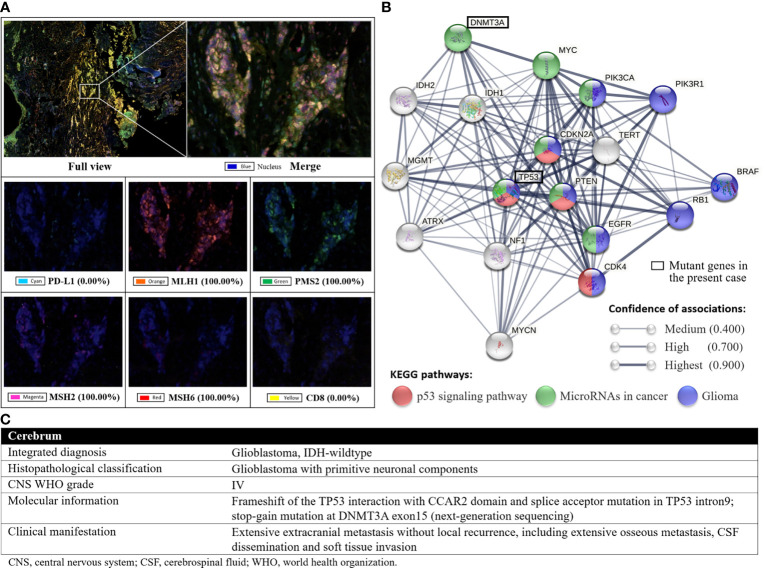
**(A)**, the results of the multiplex immunohistochemistry with fluorescence imaging. The positive rates of MLH1, PMS2, MSH2 and MSH6, the four main proteins involved in the DNA mismatch repair system, were 100%, indicating proficient mismatch repair. **(B)**, the protein-protein interaction network computed by STRING v11.0 (URL: https://string-db.org/), which comprise two mutant genes in the present case and 16 frequently reported genes associated with GBM and GBM-PNC. The DNMT3A might play an important role in the epigenetic regulation of the biological behavior of GBM-PNC. **(C)**, the layered report of the confirmed diagnosis according to the 2021 WHO Classification of Tumors of the CNS (DOI: 10.1093/neuonc/noab106).

The molecular diagnosis of the primary lesion revealed isocitrate dehydrogenase (IDH) wild type (IDH1^R132^ and IDH2^R172^), no mutation in the TERT promoter or BRAF^V600E^, no chromosome arm 1p/19q co-deletion, and a lack of MGMT promoter methylation, which suggests primary GBM with poor prognosis. The genomic profiling of tumor tissues from cervical spine metastases (a glioma-specific 131-gene and 4- chromosome panel) identified pathogenic mutations in TP53 and potentially damaging mutations in other genes (**Table 1**, above TP53). Based on whole-exome sequencing, the tumor mutation burden of thoracolumbar metastatic lesions from the third operation was calculated to be 0.49 muts/Mb, and no microsatellite instability was detected. The multiplex immunohistochemistry/immunofluorescence identified proficient mismatch repair status (**Figure 7A**). These findings collectively indicated a poor response to the immune checkpoint inhibitor, and hence, anti-PD-1/PD-L1 therapy was not considered (13). A concurrent 825 tumor-related gene panel for thoracolumbar metastases revealed several other likely pathogenic variants (**Table 1**, ANTXR1 and below), among which DNMT3A is predicted to be an epigenetic driver of high malignancy and dismal prognosis (**Figure 7B**) (14). According to the latest 2021 WHO Classification of Tumors of the CNS (15), the confirmed diagnosis of GBM-PNC was established after synthesizing the clinical, histological, and genetic findings (**Figure 7C**).

**Table 1:** The likely pathogenic variants identified in cervical spine and thoracolumbar metastases.

Genes	Variants	Abundance	Clinical significance*	Protein function (dbSNP Reference SNP ID)	Pathogenicity prediction	
					SIFT^#^ (score)	Polyphen2^$^ (score)
BCOR	exon10; missense p.D1420N; c.4258G>A	99.54%	Tier III	Unknown	Deleterious (0.033)	Possibly damaging (0.927)
DNMT3A^&^	exon15; stop gained p.R598*; c.1792C>T	49.93%-55.0%	Tier III	Loss of function (rs568207978)	N/A	N/A
MPL	exon7; missense p.A371V; c.1112C>T	48.74%	Tier III	Unknown	Deleterious (0.05)	Probably damaging (0.999)
TP53^&^	exon5; frameshift p.R158Pfs*23; c.472dupC	35.5%-40.05%	Tier II	Loss of function	N/A	N/A
ACVR1	exon8; missense p.A333T; c.997G>A	37.09%	Tier III	Unknown	Deleterious (0)	Probably damaging (0.993)
TP53^&^	intron9; splice acceptor c.994-1G>A	20.48%-24.8%	Tier II	Loss of function (rs587782272)	N/A	N/A
ANTXR1	exon9; missense p.I221P; c.661A>T	50.5%	Tier III	Unknown	Deleterious (0)	Probably damaging (0.995)
VNN2	exon1; frameshift p.S25AfsTer6; c.74_78del	32.8%	Tier III	Loss of function	N/A	N/A
INSL4	exon2, missense p.P87L; c.260C>T	30.6%	Tier III	Unknown	Deleterious (0.01)	Possibly damaging (0.636)
BAZ2A	exon10; missense p.G672A; c.2015G>A	30.0%	Tier III	Unknown	Deleterious (0.02)	Probably damaging (0.999)
TLR8	exon2; missense p.A959D; c.2876C>A	27.5%	Tier III	Unknown	Deleterious (0)	Probably damaging (1)

N/A, not available. *Classification was made according to the “Standards and Guidelines for the Interpretation and Reporting of Sequence Variants in Cancer” (DOI: 10.1016/j.jmoldx.2016.10.002). ^#^Refers to the ‘Sorting Tolerant from Intolerant’ algorithm (URL: http://provean.jcvi.org/genome_submit_2.php?species=human). ^$^Refers to the software tool, PolyPhen-2 (URL: http://genetics.bwh.harvard.edu/pph2/). ^&^Mutations identified in both gene tests of cervical metastases (the second operation) and thoracolumbar metastases (the third operation).

## Discussion

In this report, we focused on the unusual extensive osteophilic extracranial metastases from GBM-PNC, a rare concomitance of two highly malignant CNS tumor components, glioblastoma and PNC (previously called PNET). Given its rarity, the diagnosis, treatment, and investigation of GBM-PNC are difficult. Since the awareness of this uncommon entity, around 200 cases of GBM-PNC have been reported, of which many were sporadic (6, 7, 16–18). To our knowledge, this is the first report of extensive skeletal metastases from GBM-PNC with complete information about multiple surgical interventions for both intracranial and spinal lesions. The present case adds practical information about the treatment and outcome of this challenging GBM subtype.

Studies on composite CNS tumors with malignant glial and neuronal elements can be traced back to 1973, when the nomenclature of “primitive neuroectodermal tumor” was proposed by Hart and Earle (19, 20). However, early reports adopted an unfocused concept, the “malignant glioneuronal tumors” (MGNT), with pathological features of the glial component resembling astrocytoma or other lower-grade gliomas instead of GBM in most cases (19). Varlet et al. reported 40 cases of MGNT, of which five had PNET-like foci, and only two were confirmed as GBM-PNC (21). In 2001, Wharton et al. first identified primitive neuroepithelial components in gliosarcoma, a variant of GBM, making it the first report of GBM-PNC in broad terms (22). The first case series was published in 2009 by Perry and colleagues (7). The 53 enrolled patients were defined as CNS malignant glioma with PNET components, including 47 GBM-PNCs. Highly aggressive biology, 9.1-month median survival, anaplastic hypercellular PNC, up to 40% of cerebrospinal fluid (CSF) dissemination, and possible application of platinum-based chemotherapy were reported by the authors. The latest and largest cohort was studied by Suwala et al., who reported a distinct methylation profile with frequent alterations of TP53 in 63 cases of GBM-PNC (17).

Extracranial metastases are rare, seen in only 0.4%–2% of GBMs (3, 4, 23, 24). The low incidence of extracranial metastases is associated with the short survival and intrinsic blood–brain barrier (2). Recently, a growing number of related cases and the detection of circulating GBM tumor cells have confirmed the ability of GBM to develop extracranial metastases (25, 26). Bearing in mind inconsistencies in time-frame and inclusion criteria, previous literature reviews identified 79–150 cases of extracranial or extra-CNS metastases from GBM, and the most prevalent extracranial metastases sites were bone, lung, lymph nodes, and liver (2, 25, 27–30). Extracranial metastases of GBM were also reported in pediatric patients and cases with the absence of previous neurosurgical intervention (31–33). Goodwin and colleagues reviewed 28 cases of GBM metastases to the vertebra, with a mean age at presentation of 38.4 years and an average overall survival of 26 months, of which seven patients received surgery for vertebral metastases (34). After a systematic search on Medline, Embase, and Google Scholar, we retrieved 25 cases of GBM-PNC with extracranial metastases from 14 reports (**Table 2**) (7, 16, 17, 22, 35–44). CSF dissemination occurred in 88% (22/25) of the patients, whereas only 4 patients (16%) suffered bone metastases. The age at diagnosis and overall survival ranged from 17 to 65 years and 2 to 31 months, respectively.

**Table 2:** Reported cases of extracranial metastasis of glioblastoma multiforme with primitive neuronal component.

First author and year of publication	Number of cases	Article type	Age/median age at diagnosis	Gender	Location of the primary tumor	Intracranial recurrence/metastasis	Location of extracranial metastasis	Intervention	Survival after initial diagnosis (months)
Suwala, 2021 (17)	4	Case series	N/A	N/A	N/A	N/A	Leptomeningeal dissemination with spinal metastasis in four	N/A	N/A
Maekawa, 2021 (35)	1	Case report	65	Male	right temporal lobe	Yes	Multiple osseous metastases to the spine, pelvis, bilateral humerus and femur Lymph nodes (right neck, mediastinum, pulmonary hilum, and para-aorta) Liver Visceral and parietal Pleura	Brain surgery (not specified)	2
Donabedian, 2021 (16)	1	Case report	52	Female	left frontal lobe	Yes	Lung	GTR+CRT+ Pembrolizumab+ transcranial electric field generator for intracranial lesion	7
Kay, 2020 (36)	1	Image	17	Female	left temporal lobe	N/A	Multiple leptomeningeal drops and vertebral metastases of the spine Right humerus Pelvis Peritoneal seeding *via* a ventriculoperitoneal shunt	Brain surgery (not specified)	N/A
Tamai, 2019 (37)	1	Case report	49	Male	right temporal lobe	Yes	Whole spinal canal (multiple meningeal seeding) Lung	GTR+CRT+TMZ for intracranial lesion Surgical resection and Gamma knife surgery for cervical metastases CRT+TMZ+bevacizumab for other meningeal seeding lesions	12
Ricard, 2019 (38)	1	Case report	37	Male	right cerebellum	Yes	The spine (multiple vertebral metastases and meningeal seeding)	Repeated surgical resection, CRT, TMZ, Pembrolizumab, Optune device, gamma knife stereotactic radiosurgery, Avastin	31
Vollmer, 2019 (39)	1	Case report	47	Male	right temporal lobe	Yes	Whole spinal canal (CSF dissemination)	STR+CRT+TMZ for intracranial lesion Surgical decompression+CRT for spinal lesions	N/A
Johanns, 2016 (40)	1	Case report	31	Male	Left frontotemporal lobe	Yes	Cervical and thoracic spinal cord (C7-T2, T7-8, “drop” metastasis)	STR+CRT+TMZ for intracranial lesion Laminectomy and GTR+ Pembrolizumab and CRT for spinal metastases	N/A (>29)
Chu, 2015 (41)	1	Case report	49	Male	right temporal lobe	Yes	Sacral canal (CSF dissemination)	GTR+CRT+TMZ for intracranial lesion	28
Kimbason, 2015 (42)	1	Case report	42	Male	left frontal lobe	Yes	Not specified (CSF dissemination)	GTR+CRT+TMZ for intracranial lesion Novocure-tumor treating fields Bevacizumab with ifosfamide, carboplatin, and etoposide	24
Willard, 2015 (43)	1	Case report	29	Female	Right temporal lobe	Yes	Whole spinal cord (multifocal leptomeningeal metastases maximal in the lumbar cord and focal in the cervical cord)	Surgical resection and chemotherapy (not specified)	12
Song, 2011 (44)	2	Case series	1. 44 2. 35	Female Male	Right posterior parietal lobe Right frontal lobe	N/A N/A	Not specified (CSF dissemination) Not specified (CSF dissemination)	GTR+CRT+TMZ for intracranial lesions GTR+CRT+TMZ for intracranial lesions	Alive at 31 months Alive at 4 months
Perry, 2009 (7)	8	Case series	N/A	N/A	N/A	N/A	Not specified (CSF dissemination in eight (40%), bone marrow metastasis in one that also had CSF spread)	N/A	N/A
Wharton, 2001 (22)	1	Case report	53	Male	Left temporal lobe (gliosarcoma)	Yes	Multiple bone metastases (skull, ribs, thoracolumbar spine and pelvis) Liver	Surgical resection and chemotherapy	5

N/A, not available; CSF, cerebrospinal fluid; GTR, gross total resection; STR, subtotal resection; CRT, conformal radiotherapy; TMZ, temozolomide.

The young age at onset, hemorrhagic cystic morphology, restricted diffusion on diffusion-weighted imaging, and the Ki-67 labeling index >40% in the present case indicate co-existence of PNET-like component, which necessitate further histological screening for PNC in supposed typical GBM (7, 44–47). Grossly, the tissues of primary and metastatic lesions were all very fragile and loose, and thus prone to any accidental pinching or squashing during the surgical resection and/or specimen processing. Consequently, the micromorphology might be altered. This may be partly responsible for the more atypical geometric distribution and less clear interfaces between GBM and PNC areas in this case, compared with the well-demarcated nodular PNC within a GBM background in previous reports (7, 18, 44). An elevated proportion of PNC in metastatic lesions was observed, which suggests the invasiveness of PNC (7, 48, 49). The PNC in this case showed characteristics of both central (originating from brain parenchyma and absence of EWSR1 rearrangement) and peripheral (CD99^+^) PNET (50, 51). Peripheral PNET belongs to the Ewing sarcoma family of tumors that are common in bone and soft tissue (52). Therefore, the significant predilection of metastases for osseous tissue in this patient may be attributed to the existence of PNC.

The incidence of TP53 mutation is higher in GBM-PNC than in typical GBM (73%–80% *vs.* 25%–30%) (17, 53). Inactivation of p53 is linked to increased cell proliferation, neoplastic invasion, and a more stem-like phenotype (54). Therefore, the mutant TP53 identified in this case may account for the aggressiveness and extensive extracranial metastases. Our patient’s stop-gain mutation in DNMT3A was previously reported in an overgrowth syndrome with intellectual disability, but he did not exhibit the phenotype described by the authors (55). DNMT3A encodes DNA methyltransferase, and is frequently mutated in acute myeloid leukemia (56). This gene can also influence the prognosis of GBM by methylation of microRNAs (57). Because GBM-PNC has a unique DNA methylation profile, certain DNMT3A mutations may negatively affect the prognosis of GBM-PNC at the epigenome level, but further mechanism study is needed (14, 17).

The treatment dilemma of GBM-PNC lies in the co-occurrence of two histological components that have distinct clinicopathological features, responses to drugs, and prognoses. Hence, the therapeutic rationale for GBM-PNC is to combine the standard treatment for GBM with adequate coverage for PNC, to lower the risk of recurrence and extracranial metastases (6). Maximal resection of the intracranial lesion followed by radiotherapy is essential. The Stupp protocol (applied in the present case) and/or craniospinal irradiation with adjuvant TMZ can be selected according to the histological predominance of GBM or PNC (58, 59). If the adverse effects are tolerated, concomitant platinum−based chemotherapy for PNC should also be implemented (7, 41, 42, 46, 60, 61). In hypermutated GBM-PNCs, checkpoint blockade immunotherapy can be considered (40). In dealing with spinal metastasis, early detection can lead to a better prognosis. Therefore, in addition to brain MRI, surveillance MRI of the total spine is recommended, and a further PET/CT under the condition of positive findings on MRI, given the high risk of CSF dissemination in GBM-PNC (36, 62). A comprehensive evaluation based on the neurologic, oncologic, mechanical instability, and systemic disease (NOMS) decision framework is indispensable (63). Notably, the oncologic part refers to the predicted response to available therapies. More aggressive open surgical strategies, including corpectomy and en-bloc resection, are judicious in cases with severe spinal instability, high-grade metastatic epidural spinal cord compression, and a relatively good general status (64). Enhanced collaborations between radiation oncologists and spine surgeons are crucial for optimizing stereotactic spine radiotherapy, minimizing radiation-related wound complications, and avoiding excessive surgery (65).

The primary limitation of the present study is the limited significance and low level of evidence, which are intrinsic properties of a single case. Secondly, the genetic tests of the tissue samples from the three operations used inconsistent platforms and methods, which made it unfeasible to analyze the evolution of molecular profiles from primary to metastatic lesions. Another weakness was the lack of treatment targeting PNC in this patient. Future studies on multimodality treatment with long−term follow-up are imperative to optimize the therapeutic algorithm for GBM-PNC (6).

In conclusion, the present case study and review of the literature summarizes the clinical, histological, and genetic features of GBM-PNC and highlights the occurrence and severity of extensive extracranial metastases. This rare variant of GBM requires aggressive multimodal treatment, including surgery, standard chemoradiotherapy for typical GBM, and the early introduction of craniospinal irradiation and platinum−based chemotherapy for PNET-like components. Given the lack of clear diagnostic work-flow, the vigilance for PNC in supposed typical GBM should be kept in mind, and the pathological screening of PNC is recommended in patients with early onset and intratumoral hemorrhage to avoid diagnosis delay and facilitate timely treatment. Spine surgery for axial skeletal metastasis from GBM-PNC is appropriate in patients with chemoradioresistance and relatively good general status, with the objectives of restoring spinal stability and relieving spinal cord compression.

## Data Availability Statement

The original contributions presented in the study are included in the article. Further inquiries can be directed to the corresponding authors.

## Ethics Statement

The studies involving human participants were reviewed and approved by Institutional Review Board, Beijing, Tiantan Hospital, Capital Medical University, Beijing, China (number KY2014-025-02). The patient’s parents provided their written informed consent to participate in this study on behalf of the patient.

## Author Contributions

Conception and design: TR and BL. Administrative support: BL and WL. Provision of study materials or patients: BL, WZ, XQ, and WL. Collection and assembly of data: TR, WC, DZ, BW, and ZK. Data analysis and interpretation: TR, WZ, and BL. Manuscript writing and/or revising: All authors. All authors contributed to the article and approved the submitted version.

## Funding

The corresponding author (BL) has received funding from the National Natural Science Foundation of China (grant numbers 81772370 and 81972084), National Key Research and Development Program of China (grant number 2018YFF0301103), and Beijing Municipal Administration of Hospitals Clinical Medicine Development of Special Funding Support (grant number XMLX201803). The other authors certify that neither they, nor any member of their immediate families, have other funding or commercial associations (consultancies, stock ownership, equity interest, patent/licensing arrangements, etc.).

## Conflict of Interest

The authors declare that the research was conducted in the absence of any commercial or financial relationships that could be construed as a potential conflict of interest.

## Publisher’s Note

All claims expressed in this article are solely those of the authors and do not necessarily represent those of their affiliated organizations, or those of the publisher, the editors and the reviewers. Any product that may be evaluated in this article, or claim that may be made by its manufacturer, is not guaranteed or endorsed by the publisher.

## Abbreviations

CNS, central nervous system; CSF, cerebrospinal fluid; CT, computed tomography; GBM, glioblastoma multiforme; IDH, isocitrate dehydrogenase; MGNT, malignant glioneuronal tumors; MRI, magnetic resonance imaging; PET/CT, positron emission tomography/CT; PNC, primitive neuronal component; PNET, primitive neuroectodermal tumor; SINS, Spine Instability Neoplastic Score; Syn, synaptophysin; TMZ, temozolomide; VAS, visual analog scale.
